# Validated Near‐Infrared Spectroscopy and Chemometric Modelling for Rapid Quantification of Essential Oil Yield and α/β‐Santalol in *Santalum Album* L.

**DOI:** 10.1002/gch2.70137

**Published:** 2026-07-31

**Authors:** Muhammad Hassnain, Muhammad Rizwan Azhar

**Affiliations:** ^1^ School of Engineering Edith Cowan University (ECU) Joondalup Western Australia Australia

**Keywords:** artificial intelligence, chemometric modelling, essential oil yield, machine Learning, near‐infrared spectroscopy, portable spectroscopy, sandalwood processing, wood classification

## Abstract

The sandalwood industry remains constrained by destructive, time‐intensive assays for essential oil (EO) yield, composition, moisture content, and wood fraction, which limit real‐time decision‐making. We report a unified near‐infrared spectroscopy‐artificial intelligence (NIRS‐AI) platform for non‐destructive analytics across the *Santalum album* L. value chain. Reflectance and transmittance spectra from solid matrices (disks, logs, chips, and powders), oils, ethanol extracts, and CID‐derived emulsions were acquired using benchtop (400–2500 nm) and portable (900–1700 nm) spectrometers and calibrated against hydrodistillation, gas chromatography, extraction, and moisture assays. Advanced chemometric modelling using AI‐based machine learning techniques, including regularised regression, ensemble learning, boosting algorithms, and neural networks, was used to capture nonlinear spectral–property relationships and benchmark application‐specific predictive performance. Independent external validation yielded *R* ^2^ values of 0.97 for EO yield, 0.96 and 0.94 for α‐ and β‐santalol, 0.99 for the heartwood‐sapwood ratio, 0.86 for oil moisture, 0.98 for ethanol extract yield, and 0.94 for portable emulsion‐yield prediction. NIRS also outperformed visible spectra for classifying heartwood, sapwood, inner and outer bark, and transition wood. Δ*R* ^2^ analysis showed that ensemble models were comparatively robust to preprocessing variation, whereas linear models were more sensitive to changes in the variance structure.

## Graphical abstract

Near‐infrared spectroscopy enables rapid, non‐destructive quality assessment across diverse sample matrices. Combined with conventional chemometrics and AI/ML models, it provides accurate prediction of key quality attributes, supporting routine quality control, process optimisation, and real‐time industrial decision‐making.

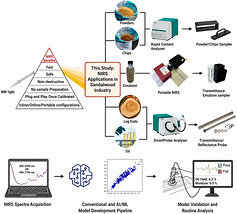

## 1 Introduction

Indian Sandalwood (*Santalum album* L. or *S. album*) is one of the most valuable aromatic woody biomaterials in the world, occupying a unique position at the intersection of luxury fragrance chemistry, traditional medicine, cosmetics, and high‐value natural product trade [1]. Its economic value derives almost entirely from the essential oil (EO) concentrated in the heartwood (HW), whose olfactory quality and market price are governed primarily by the abundance of sesquiterpene alcohols, particularly α‐ and β‐santalol [2, 3]. These compounds define not only sensory properties but also regulatory standards and commercial grading [4]. Consequently, accurate quantification of oil yield and chemical composition is not merely an analytical task; it is the foundation of valuation, trade transparency, and quality assurance across the global sandalwood supply chain [5, 6].

**FIGURE 1:**
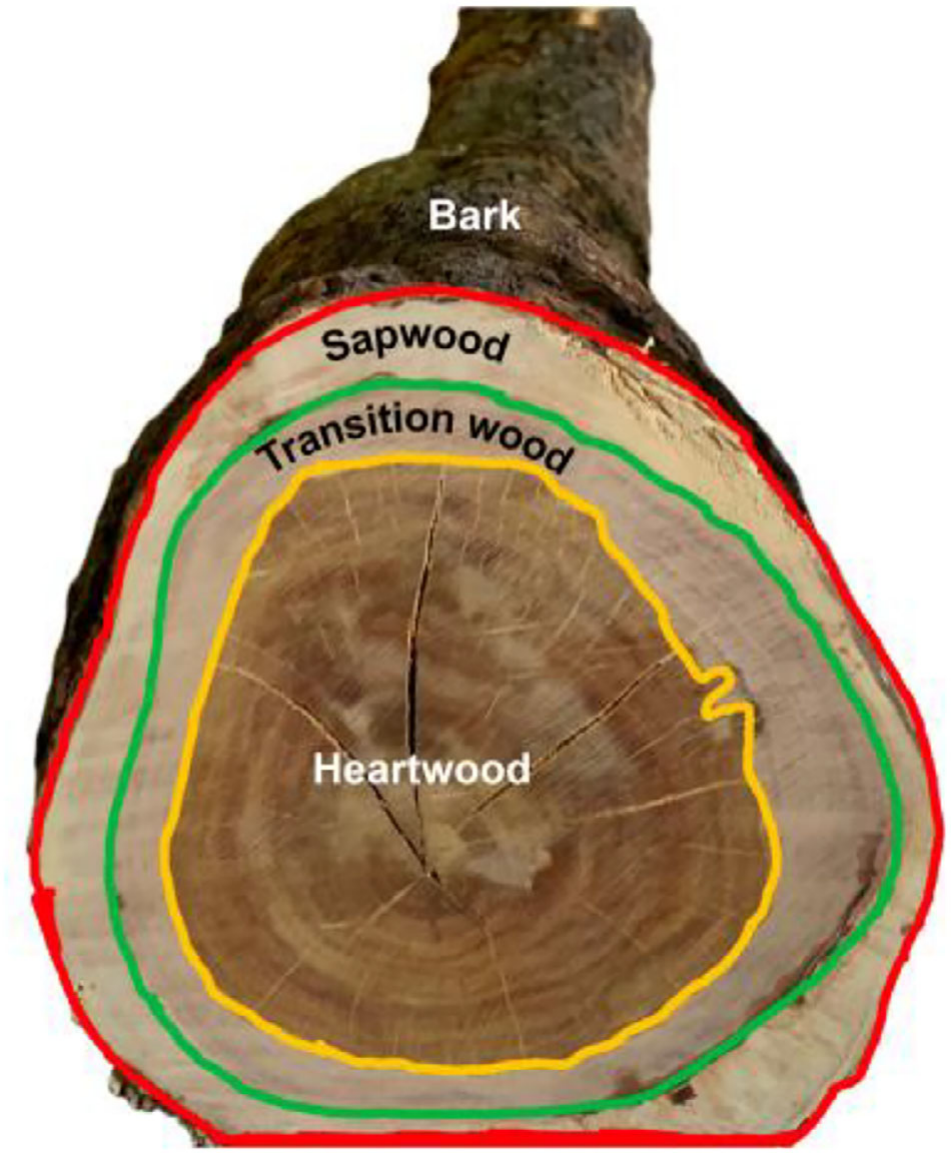
Radial organisation of woody stem tissues in a mature *S. album* tree trunk. Concentric layers show protective bark, conductive SW, a transition zone, and dense, non‐conductive HW formed through progressive xylem aging.

The localisation of these oil‐rich extractives is intrinsically linked to the anatomical and physiological organisation of woody stems [7]. As shown in Figure 1, a mature trunk of *S. album* is radially structured into protective bark, physiologically active sapwood (SW), an intermediate transition zone, and the inner HW, which forms through progressive xylem aging [8]. Unlike SW, which is primarily involved in water transport, HW represents a metabolically inactive but chemically enriched compartment where secondary metabolites, including the sesquiterpene alcohols responsible for sandalwood fragrance, accumulate [4, 9, 10, 11]. This spatial heterogeneity underpins both the value and the analytical complexity of *S. album*, as commercially relevant chemistry is concentrated in a specific, non‐conductive wood fraction rather than being distributed uniformly throughout the stem. Consequently, representative sampling and rapid compositional assessment are essential for valuation, trade transparency, and quality assurance across the global sandalwood supply chain [5, 12].

Despite its premium status, the industrial processing of *S. album* remains constrained by analytical methodologies that have changed little over the decades [13 ^,^ 14]. Determination of oil yield relies on prolonged hydrodistillation (HD) (often 24 h), while chemical quality assessment requires gas chromatography (GC) [15]. Moisture determination, wood fraction analysis, and pre‐distillation screening involve additional destructive, laboratory‐based procedures. Although accurate, these approaches are slow, labour‐intensive, and incompatible with real‐time or large‐scale industrial decision‐making [16]. As a result, critical operational decisions, including harvest valuation, batch consolidation, distillation control, and quality grading, are often made with incomplete analytical information, leading to energy inefficiency, yield losses, increased process variability, and avoidable waste [17, 18]. This mismatch between material value and analytical speed represents a fundamental bottleneck in the modern sandalwood industry [13].

The need for rapid analytics is intensified by broader market pressures [19]. The scarcity and high value of *S. album* create incentives for adulteration, substitution, and mislabelling, while increasing emphasis on sustainability and traceability demands more transparent, verifiable quality assessment [20, 21]. Reliable, non‐destructive, and deployable analytical tools are therefore required not only for process optimisation but also for authenticity verification and regulatory compliance [22]. Addressing these challenges requires a shift from episodic laboratory testing to continuous, data‐driven material intelligence [23].

Near‐infrared spectroscopy (NIRS) offers a compelling technological pathway toward this transformation [24]. NIRS probes overtone and combination vibrations of C─H, O─H, and N─H functional groups, which dominate both lignocellulosic matrices and the sesquiterpene‐rich extractives characteristic of *S. album* HW [25]. The broad 400–2500 nm spectral window captures chemically informative absorption features associated with hydrocarbons and alcohols, while the 900–1700 nm region enables compact, field‐deployable instrumentation suitable for in‐line or at‐line monitoring. In principle, these spectral domains encode information about oil‐bearing extractives, wood polymers, and moisture, enabling simultaneous inference of quantity and quality from rapid reflectance measurements [26, 27].

However, translating this potential into industrially reliable analytics is non‐trivial [28]. NIR spectra of wood and complex liquids are characterised by overlapping absorption bands, strong multicollinearity, and significant scattering effects arising from heterogeneous structure, particle size, and phase interactions [29]. Traditional linear chemometric approaches, such as PCA and PLS regression, provide useful dimensionality reduction but often fail to fully capture nonlinear relationships between spectral features and target variables, particularly in heterogeneous solid matrices and multiphase systems [30, 31].

Recent advances in artificial intelligence (AI) and machine learning (ML) provide a methodological foundation for overcoming these limitations [32]. Algorithms such as support vector machines, ensemble tree methods, gradient boosting, and deep neural networks can model high‐dimensional nonlinear relationships and uncover predictive structures that are inaccessible to linear projections [33, 34]. When coupled with rigorous spectral preprocessing, derivative transformations, scatter correction, smoothing, and feature selection, AI‐enhanced NIRS can achieve very high predictive performance for complex biological materials, including species discrimination, yield estimation, and compositional analysis [35].

Yet, despite growing interest, the application of NIRS to *S. album* processing remains fragmented [26]. Existing studies typically focus on a single matrix (solid wood or oil), rely heavily on linear models, lack systematic benchmarking against modern nonlinear approaches, and seldom include independent external validation [32]. Portable NIRS systems, critical for in‐field and in‐process deployment, have received insufficient attention [36]. Most importantly, prior work rarely adopts a process‐level perspective that links spectral predictions directly to industrial decisions such as distillation optimisation, feedstock segregation, or automated material sorting [37]. No study has yet to establish a unified analytical framework capable of addressing the full diversity of *S. album* matrices, including intact disks, logs, chips, powders, pure oils, solvent extracts, and process‐derived emulsions, within a single NIRS‐AI architecture.

This multidimensionality is not incidental but intrinsic to the economics of sandalwood. Oil yield, chemical composition, moisture content, and HW‐SW ratio are only partially correlated; high‐yield material does not necessarily exhibit superior santalol profiles, and wood‐fraction composition governs both extractive availability and process efficiency [38]. Each matrix presents distinct optical and chemical challenges, from scattering‐dominated solids to solvent‐rich extracts and turbid emulsions [39, 40]. An effective analytical solution must therefore operate across physical states and processing stages, providing coherent intelligence rather than isolated predictions [41].

To operationalise this requirement, the present study implements a unified analytical framework that couples near‐infrared spectroscopy with advanced ML across all industrially relevant matrices of *S. album*. The methodological design was conceived not as a collection of independent models, but as an integrated platform architecture in which sampling strategy, spectral acquisition, preprocessing, feature engineering, and model validation form a continuous pipeline.

## 2 Materials and Methods

### 2.1 Study Design and Analytical Framework

**FIGURE 2:**
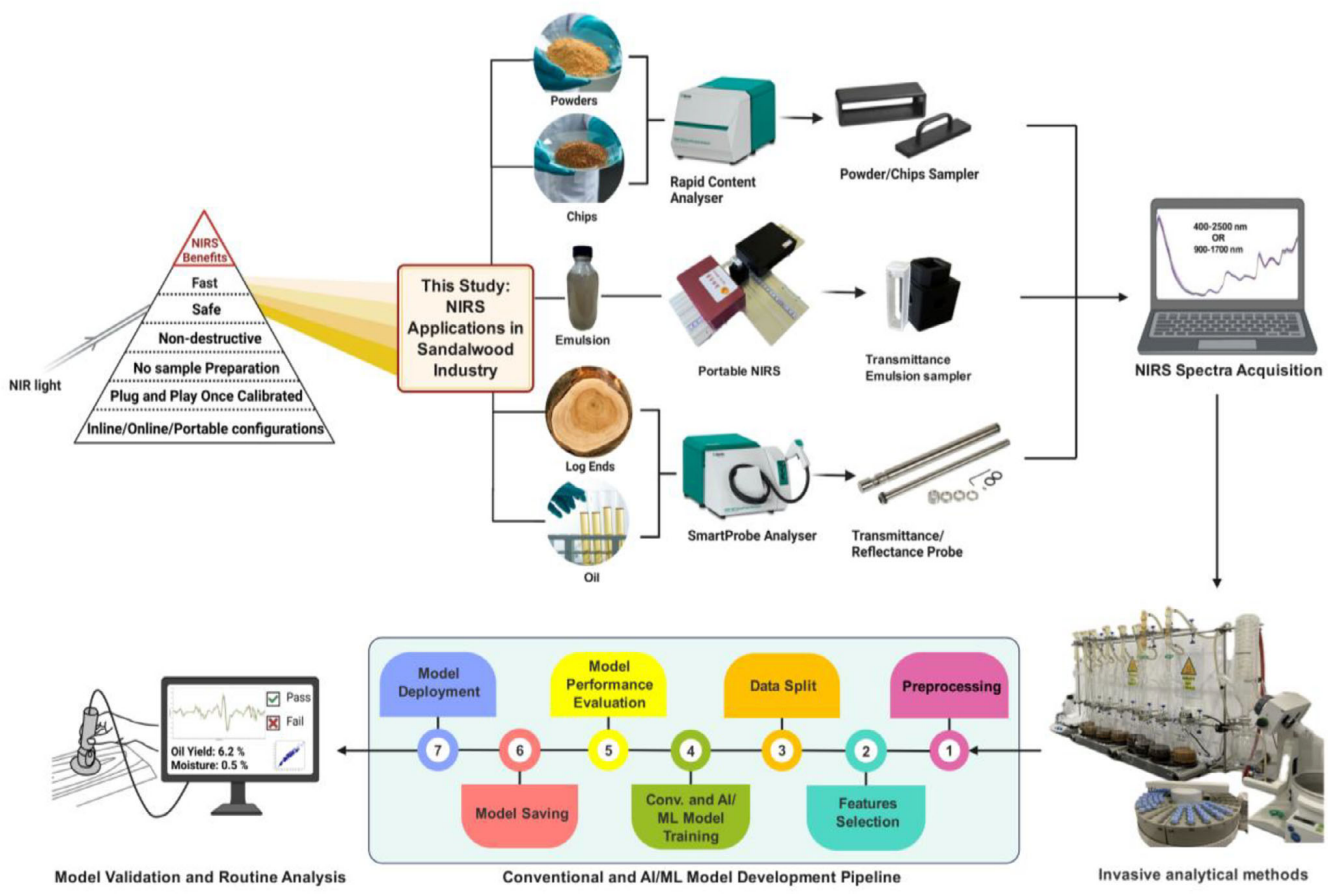
Conceptual overview of the NIRS‐AI framework for end‐to‐end qualitative and quantitative analytics in sandalwood processing. The schematic shows the investigated matrices (log ends, chips, powders, oils, solvent extracts, and CID‐derived emulsions), the benchtop (400–2500 nm) and portable (900–1700 nm) instruments, and the unified chemometric and machine‐learning pipeline.

This investigation was conducted as a systems‐level analytical study of industrially relevant *S. album* matrices, aimed at replacing time‐intensive destructive assays with a deployable NIRS‐AI platform. Rather than addressing yield, chemical composition, moisture, extraction behaviour, and wood‐type classification as independent problems, all analytical tasks were embedded within a unified workflow spanning spectral acquisition, preprocessing, feature engineering, model training, and validation [42]. The end‐to‐end architecture of this framework is illustrated in Figure 2, which links heterogeneous sample matrices (solids, powders, oils, extracts, and emulsions) to benchtop and portable NIRS systems and a harmonised chemometric‐machine learning pipeline. This structure reflects the industrial need for a single analytical backbone adaptable to at‐line, in‐line, and field‐deployment contexts.

A summary of modelling configurations across all applications, including matrix type, reference method, instrument, spectral range, preprocessing strategy, algorithm class, and dataset partitions, is provided in Table .

**TABLE 1:** NIRS‐AI applications, reference methods, instrumentation, preprocessing, models, and dataset partitions. Dataset sizes are reported as spectral rows x wavelength variables; spectra acquired from a single physical sample were retained in a single partition.

Application	Matrix Type	Reference Invasive Method	Spectrometer	Spectral Range (nm)	Preprocessing Strategy	Model	Calibration set (spectra x variables)	Internal‐test set (spectra x variables)	External‐validation set (spectra x variables)
Essential oil yield	Solid wood (chips/logs/disks)	24 h HD	MBI‐NIRS	400–2500	SNV + first derivative	Hybrid (LR + Ridge)	3000 × 4201	295 × 4201	100 × 4201
α‐ & β‐santalol prediction	Oil & solid matrices	GC	MBI‐NIRS	400–2500	SNV + first derivative	Ridge	3000 × 4201	295 × 4201	100 × 4201
Powder HW‐SW ratio	Powders	Gravimetric mixture design	MBI‐NIRS	400–2500	SNV + first derivative	Hybrid (LR + Ridge)	1100 × 4201	100 × 4201	50 × 4201
Moisture in oil	Liquid oil	LMA	MBI‐NIRS	400‐2500	SNV	AdaBoost	1500 × 4201	120 × 4201	66 × 4201
Ethanol extract yield	Liquid extract	Gravimetric post‐extraction	MBI‐NIRS	400–2500	SNV	Ridge	400 × 4201	50 × 4201	20 × 4201
CID emulsion yield	Oil‐water emulsion	5 h HD	IRK‐NIRS	900–1700	SNV + second derivative	Deep MLP	2800 × 228	287 × 228	111 × 228
Wood‐type classification (HW/SW/BI/BO/TW)	Chips/disks	Visual + anatomical ground truth	MBI‐NIRS	400–2500	SNV + second derivative	AdaBoost	32376 × 4201	3237 × 4201	1500 × 4201

### 2.2 Materials and Sample Matrices

All experiments were conducted using *S. album* L. materials supplied by Quintis Sandalwood Pty Ltd. and harvested between 2015 and 2024. Solid matrices included disks, billets, logs, log ends, industrial and traditional Chinese medicine (TCM)‐grade chips (<5 mm), shavings, powders, pure HW, pure SW, inner bark (BI), outer bark (BO), and visually identified transition wood (TW). The material collection encompassed multiple trees and processing batches to capture biological and anatomical variability. Disk surfaces were lightly abraded before scanning to minimise surface artefacts, and replicate spectra were acquired at multiple spatial positions and linked to a unique physical‐sample identifier.

Powder samples for HW‐SW ratio modelling were prepared by milling pure HW and SW fractions and recombining them gravimetrically across the full 0%–100% SW range. Liquid matrices comprised pure hydrodistilled oils, redistilled fractions with varying moisture contents, ethanol extracts, and oil‐water emulsions produced by the instantaneous controlled pressure‐drop (CID) distillation process.

### 2.3 Reference Measurements

Quantitative targets were obtained using established laboratory methods. Essential oil yields from solid matrices and emulsions were determined by HD [43]. Chemical composition (α‐ and β‐santalol) was determined using GC, where applicable [44]. Moisture in pure oil was measured using a calibrated laboratory moisture analyser (LMA) [45]. Gravimetric mixture design defined reference values for powder HW‐SW ratios. These destructive or laboratory assays provided the ground truth for NIRS model calibration.

### 2.4 NIR Spectral Acquisition

Reflectance and transmittance spectra were collected using two complementary platforms.

#### 2.4.1 Benchtop NIRS (400–2500 nm, 0.5 nm Resolution)

Metrohm benchtop instrumentation (MBI) was used in complementary configurations. A SmartProbe was used to measure reflectance of logs and billets, while transmittance was measured for oils and extracts; a Rapid Content Analyser (RCA) was used for chips and powders. Together, these configurations provided broad VIS‐NIR‐SWIR coverage of overtone and combination bands associated with C─H and O─H vibrations in wood polymers, sesquiterpenes, and moisture [46, 47]. Chips and powders were scanned using a sliding sample holder to reduce orientation effects, whereas liquids were measured in fixed‐path‐length quartz cuvettes under temperature‐equilibrated conditions.

#### 2.4.2 Portable NIRS (900–1700 nm, 3.5 nm Resolution)

An Isuzu Optics NIRez research kit (IRK) incorporating a handheld NIR spectrometer was used for field‐ and process‐relevant measurements, particularly of CID‐derived emulsions, for which portability and real‐time deployment were essential [48].

### 2.5 Spectral Preprocessing and Feature Engineering

Because NIR spectra reflect both chemical absorption and physical scattering, preprocessing was treated as a controlled modelling variable. Evaluated transformations primarily included standard normal variate (SNV) and first‐ or second‐derivative transformations with smoothing and baseline correction. Derivative preprocessing enhanced the resolution of overlapping bands and suppressed baseline variability, particularly in extractive‐sensitive regions [49].

Feature engineering incorporated correlation‐based wavelength screening, threshold‐controlled pruning, and model‐driven importance ranking. This strategy reduced dimensionality and collinearity while retaining chemically informative regions.

### 2.6 Conventional Chemometric and AI/ML Modelling

Regression tasks included predicting EO yield (solid matrices, extracts, emulsions), α‐ and β‐santalol concentrations, moisture content in oil, and the HW‐SW ratio in powders. Classification tasks involved discriminating among HW, SW, BI, BO, and TW. Algorithms evaluated included linear and regularised regression, PLS regression, ensemble tree‐based models, boosting algorithms, and neural networks. Model families were benchmarked using identical data partitions to enable cross‐application comparisons.

### 2.7 Data Partitioning and Validation

For each application, data were divided into calibration (training), internal test, and independent external validation subsets (Table 1). The physical sample, rather than the individual spectral row, was the unit of grouping. All repeat scans and spectra collected at multiple positions on a given specimen were linked by a unique sample identifier and assigned to a single subset; consequently, no physical sample contributed spectra to more than one partition. Sample‐grouped cross‐validation within the calibration set guided model optimisation, and preprocessing and feature‐selection parameters were estimated from the calibration data only, then applied unchanged to the internal‐test and external‐validation sets. Outliers were identified using leverage and residual diagnostics. Performance was assessed using the coefficient of determination (*R* ^2^), root mean square error (RMSE), mean absolute error (MAE), and classification accuracy, as appropriate.

External‐validation subsets were withheld before model development and comprised physical specimens not used in calibration or internal testing. The broader collection spanned the 2015–2024 harvest period and included multiple trees and processing batches. Because harvest year, plantation, tree, and processing batch were not available as consistent stratification variables across all applications, the validation is reported as independent sample‐level external validation rather than as a formal cross‐year, cross‐plantation, cross‐tree, or cross‐batch transfer test.

### 2.8 Δ*R* ^2^ Analysis of Preprocessing and Feature‐Selection Effects

To quantify the effects of preprocessing and feature selection, marginal performance shifts were computed using Δ*R* ^2^, as defined in Equation (1).

(1) $$\Delta \;R_{z}^{2}=E_{m\in M:z\left(m\right)=1}\;\left[R^{2}\left(m\right)]-E_{m\in M:z\left(m\right)=0}[R^{2}\left(m\right)\right]$$

where *R* ^2^(m) denotes the test‐set *R* ^2^ for model configuration m, and M is the set of all evaluated configurations. The function z(m) is a binary indicator equal to 1 when the preprocessing component or modelling choice z is included in configuration m and 0 otherwise. The expectation operator E_m__∈M:z(m)_ = k[*R* ^2^(m)] denotes the mean over configurations satisfying z(m) = k. Thus, Δ*R* ^2^z quantifies the mean change in predictive performance attributable to component z across all evaluated configurations, providing an interpretable measure of model robustness to preprocessing variation.

### 2.9 Reproducibility and Deployment Considerations

Sampling protocols incorporated multi‐point averaging for heterogeneous materials. Calibration validity depends on consistency between the acquisition conditions used during model development and those used during deployment [50]. Portable NIRS measurements demonstrated feasibility under scattering‐dominated industrial conditions, particularly for CID emulsions, supporting translation of the framework to real‐time process environments [51].

## 3 Results and Discussion

The analytical workflow linking heterogeneous *S. album* matrices to benchtop and portable NIRS platforms within a unified chemometric‐machine‐learning pipeline is shown in Figure 2. Building on this framework, the Results and Discussion address (i) the chemical and physical basis of spectral sensitivity, (ii) dataset complexity, (iii) wavelength‐target relationships, (iv) application‐specific model performance, (v) preprocessing and model robustness, and (vi) cross‐application implications. This structure allows predictive performance to be interpreted in terms of mechanistic plausibility rather than as purely empirical pattern recognition.

### 3.1 Spectral‐Chemical Basis of Sandalwood Yield and Quality Prediction

**FIGURE 3:**
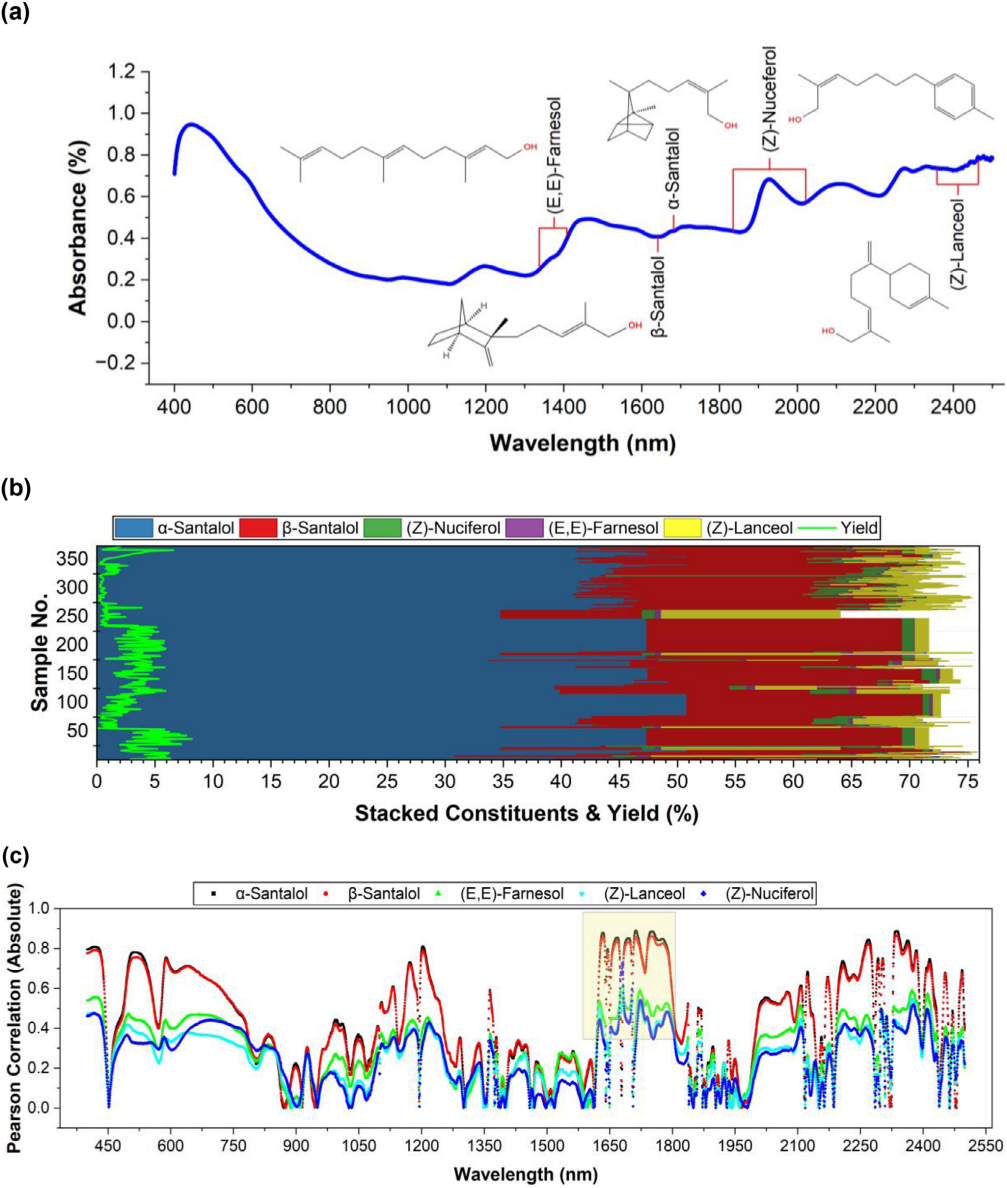
(a) Full‐range NIR absorbance spectrum (400–2500 nm) annotated with representative sesquiterpenes and sesquiterpene alcohols; highlighted regions indicate C─H and O─H overtone and combination bands relevant to yield and composition. (b) Sample‐wise EO yield and constituent profiles, showing heterogeneity and partial decoupling between yield and chemistry. (c) Absolute Pearson correlations between absorbance and reference analytes, identifying wavelength regions used by the multivariate models.

An important requirement in spectroscopic modelling is to show that predictive outcomes arise from chemically plausible spectral relationships rather than from “black‐box” coincidence [52]. Figure 3a illustrates this rationale by presenting a representative full‐range absorbance spectrum (400–2500 nm) of sandalwood HW alongside the molecular structures of α‐santalol, β‐santalol, (Z)‐nuciferol, (E,E)‐farnesol, and (Z)‐lanceol. The highlighted NIR windows contain C─H and O─H overtone and combination vibrations that contribute to absorbance. Hydroxyl‐bearing constituents, such as the santalols, contribute strongly to O─H‐related bands, providing a chemical basis for compositional models, whereas the hydrocarbon skeletons of sesquiterpenes contribute C─H‐driven signatures relevant to oil yield and extractive content. Figure 3a therefore provides a mechanistic basis for using NIRS to estimate both oil quantity and santalol composition.

### 3.2 Dataset Structure and Need for Multitarget Modelling

Although many studies reduce “quality” to a single metric, sandalwood value depends simultaneously on yield and multiple constituent fractions. Figure 3b presents sample‐wise stacked constituent profiles with an overlaid yield trace and reveals two important features:

1. **Substantial inter‐sample heterogeneity** in both yield and composition, reflecting biological and process variability.

2. **Partial decoupling** between yield and constituent distribution; for example, a high yield does not necessarily imply a high α/β‐santalol fraction.

This decoupling explains the necessity of a multi‐application analytical strategy. If yield were a reliable proxy for chemical quality, composition modelling based on GC reference data would be redundant. Instead, the results show that yield and constituent distribution occupy a multidimensional space in which material value cannot be captured by a single parameter. Independent regression targets are therefore required, and, in practice, a portfolio of models must be optimised for distinct decision layers, including harvest valuation, batch blending, distillation control, and quality assurance.

The analysis is also consistent with industry practice, in which α‐ and β‐santalol fractions are primary quality indicators. Premium sandalwood oil typically contains 60–80% combined α‐ and β‐santalols, making these constituents central to commercial grading [8]. Their separate prediction, therefore, reflects both chemical relevance and economic significance, and ensures that the spectroscopic framework addresses the metrics that define market value.

### 3.3 Spectral‐Target Correlations and Feature Relevance

Although correlation does not replace multivariate modelling, it is useful for identifying wavelength regions that are consistently relevant across targets. Figure 3c shows the absolute Pearson correlation spectra for the major constituents and yield across 400–2500 nm. Several high‐correlation windows align with expected C─H and O─H absorption domains, reinforcing chemical plausibility. The strongest correlations occur in the 1650–1800 nm range, underscoring the importance of this region for model performance. Importantly, the correlation profiles differ among yield and the individual constituents, confirming that the models capture distinct chemical phenomena rather than a single latent factor.

### 3.4 Multitarget Prediction Across Sandalwood Matrices

#### 3.4.1 Application I: EO Yield Prediction From Disks, Logs, and Chips

Accurate prediction of EO yield from intact Sandalwood matrices represents the most industrially consequential quantitative application of NIRS, as it directly influences harvest valuation, batch segregation, and distillation scheduling. This application is inherently challenging due to the heterogeneous anatomical structure of wood, which introduces strong scattering effects, baseline variability, and non‐uniform optical path lengths.

**FIGURE 4:**
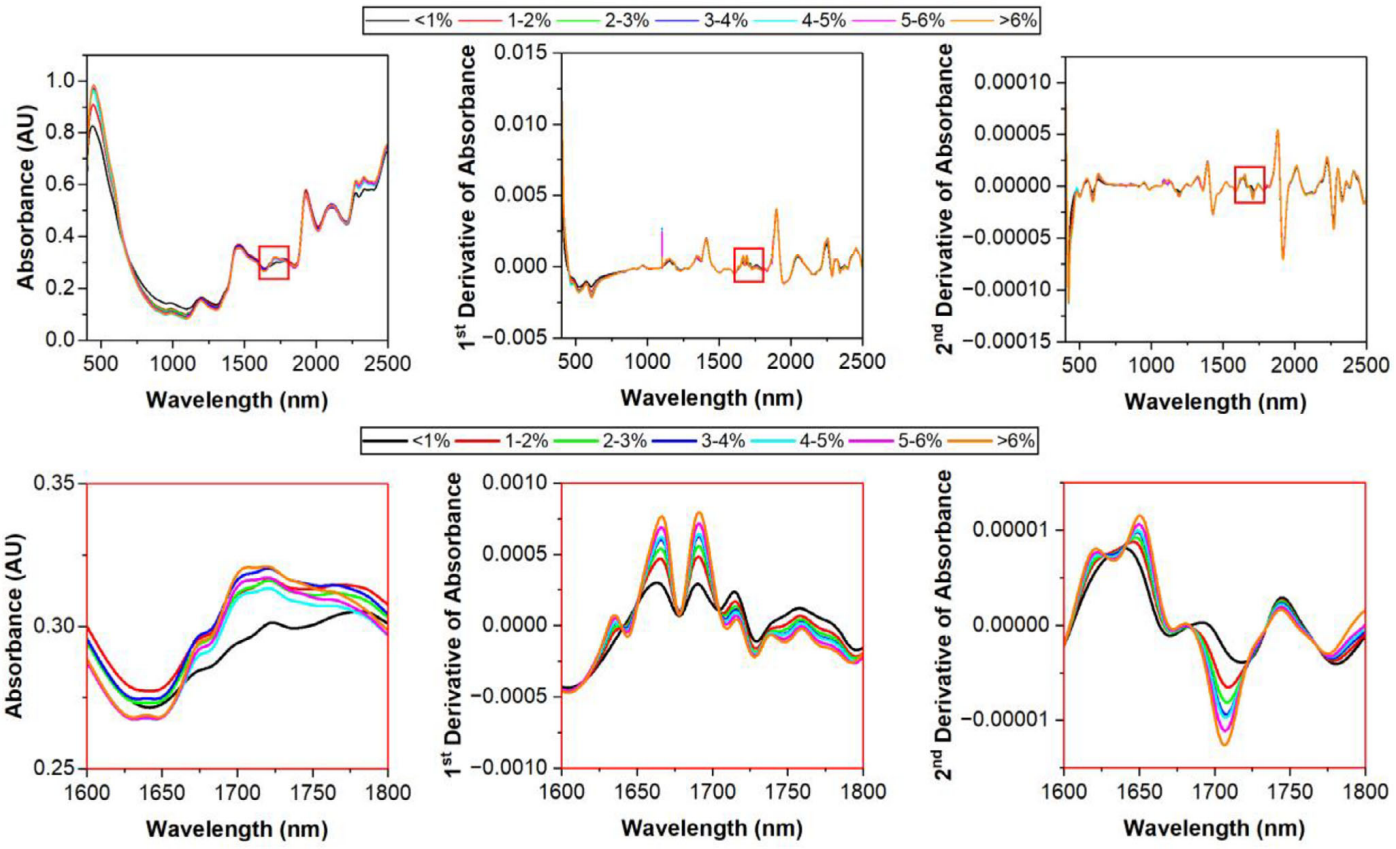
Raw, first‐derivative, and second‐derivative NIR spectra of sandalwood grouped by EO yield class (<1% to >6%). Insets show the 1600–1800 nm region, where derivative preprocessing enhances yield‐dependent spectral divergence while reducing baseline and scattering effects.

The feasibility of yield prediction is first grounded in the chemical‐spectral rationale established in Figure 3a, where the dominant oil‐bearing sesquiterpenes exhibit C─H‐ and O─H‐related overtone and combination bands within the NIR region. However, as illustrated in Figure 4, raw absorbance spectra grouped by yield class (<1% to >6%) show only modest visual separation, particularly in the lower‐wavelength region (<1200 nm), where scattering and matrix effects dominate.

**FIGURE 5:**
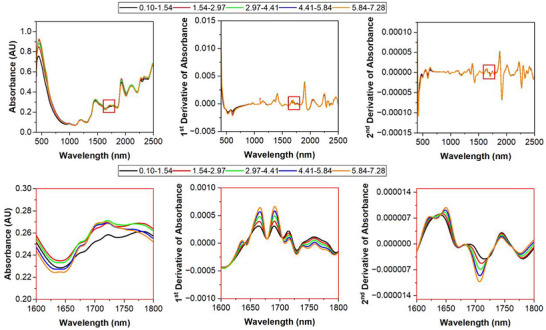
Raw and derivative NIR spectra of TCM‐grade HW grouped by EO yield class. Derivative spectra reveal systematic differences in informative wavelength regions that are not readily apparent in the raw spectra.

Derivative preprocessing plays a decisive role in resolving spectral information related to yield. As shown in Figure 4, first and second derivatives suppress baseline offsets and amplify subtle curvature changes, revealing yield‐dependent divergence in the 1600–1800 nm window (highlighted in red). This region corresponds to combination bands associated with extractive‐rich wood fractions and is therefore chemically consistent with variation in oil yield. The progressive sharpening of these features from raw to second‐derivative spectra explains the improvement observed for derivative‐based models. Figure 5 shows comparable patterns in TCM‐grade HW spectra.

The global benchmarking in Figure 14 further supports this application: linear and regularised regression, particularly linear regression and ridge regression, achieved high *R* ^2^ values for chip‐ and log‐based yield prediction. The external‐validation parity plot in Figure 15 shows close agreement between predicted and measured chip yields (*R* ^2^ = 0.97, RMSE = 0.35, MAE = 0.19), confirming generalisation to physically independent samples.

These results indicate that robust yield prediction in heterogeneous wood requires preprocessing and modelling strategies that accommodate the combined effects of scattering, absorbance, and extractive concentration.

#### 3.4.2 Application II: Α‐ and β‐Santalol Prediction

While yield determines economic volume, Sandalwood oil quality is ultimately defined by its sesquiterpene alcohol composition, particularly α‐santalol and β‐santalol. Predicting these constituents independently of yield is therefore essential for quality grading, blending strategies, and regulatory compliance.

The chemical justification for this application is explicitly visualised in Figure 3a, where the α‐ and β‐santalol molecular structures highlight the presence of hydroxyl functional groups that strongly contribute to NIR absorbance via O─H overtone and combination vibrations. However, unlike yield, differences in santalol concentration are subtle and embedded within overlapping spectral features.

**FIGURE 6:**
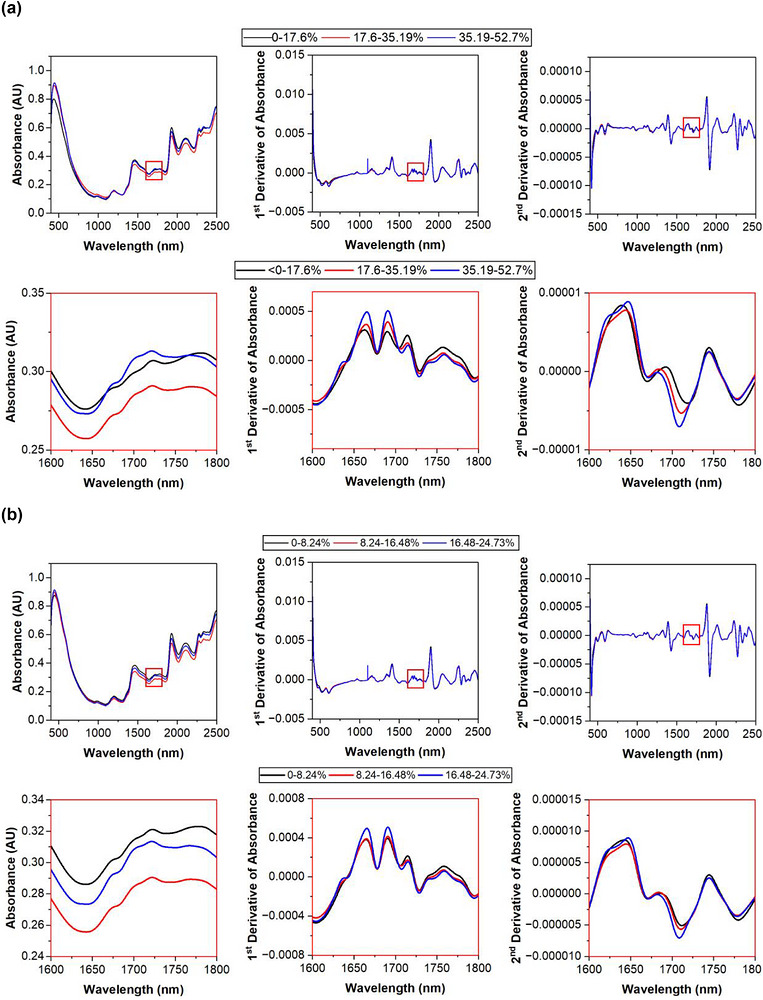
Raw and derivative NIR spectra stratified by (a) α‐santalol and (b) β‐santalol concentration ranges. Distinct derivative features in the 1650–1750 nm region correspond to O─H and C─H combination bands of sesquiterpene alcohols, providing chemically plausible inputs for compositional prediction models.

Raw spectra stratified by santalol concentration overlap substantially (Figure 6a,b), indicating that unprocessed absorbance alone is insufficient for reliable quantitative discrimination. In contrast, first‐ and second‐derivative spectra reveal systematic, concentration‐dependent structure within the 1650–1750 nm region. These derivative‐enhanced features align with alcohol‐related combination bands and provide chemically interpretable signatures associated with santalolx content.

This interpretation is reinforced by the correlation analysis in Figure 3c, which identifies strong wavelength relevance in the same NIR domain. The convergence of derivative‐resolved spectral structure and correlation‐based feature relevance confirms that the observed spectral changes are mechanistically linked to the target analytes rather than arising from incidental covariance. This chemically grounded linkage underpins the strong predictive performance of the compositional models and supports the application‐specific modelling strategies developed in this study.

The modelling rationale is further informed by the correlation analysis in Figure 3c, which reveals wavelength‐specific patterns that differ among α‐santalol, β‐santalol, and total yield. This divergence confirms that compositional prediction cannot be inferred directly from yield models and must be treated as an independent regression task. Although yield and santalol concentrations share extractive‐related absorptions, linear regression and ridge regression provided stable performance across preprocessing variations and were therefore retained for the santalol models. The external‐validation parity plots in Figure 15 yielded *R* ^2^ = 0.96 for α‐santalol and *R* ^2^ = 0.94 for β‐santalol, with low MAE and RMSE.

From an industrial standpoint, this application enables rapid screening of wood or oil before distillation or blending, reduces reliance on time‐intensive GC analysis, and may support adulteration screening and proactive quality management.

#### 3.4.3 Application III: HW‐SW Ratio Prediction in Powders

Powdered Sandalwood represents a fundamentally different analytical matrix, characterised by reduced macroscopic heterogeneity but increased sensitivity to compositional mixing ratios. The HW‐SW ratio is a critical determinant of economic efficiency, as SW contributes negligible oil yield while increasing processing and transportation costs.

**FIGURE 7:**
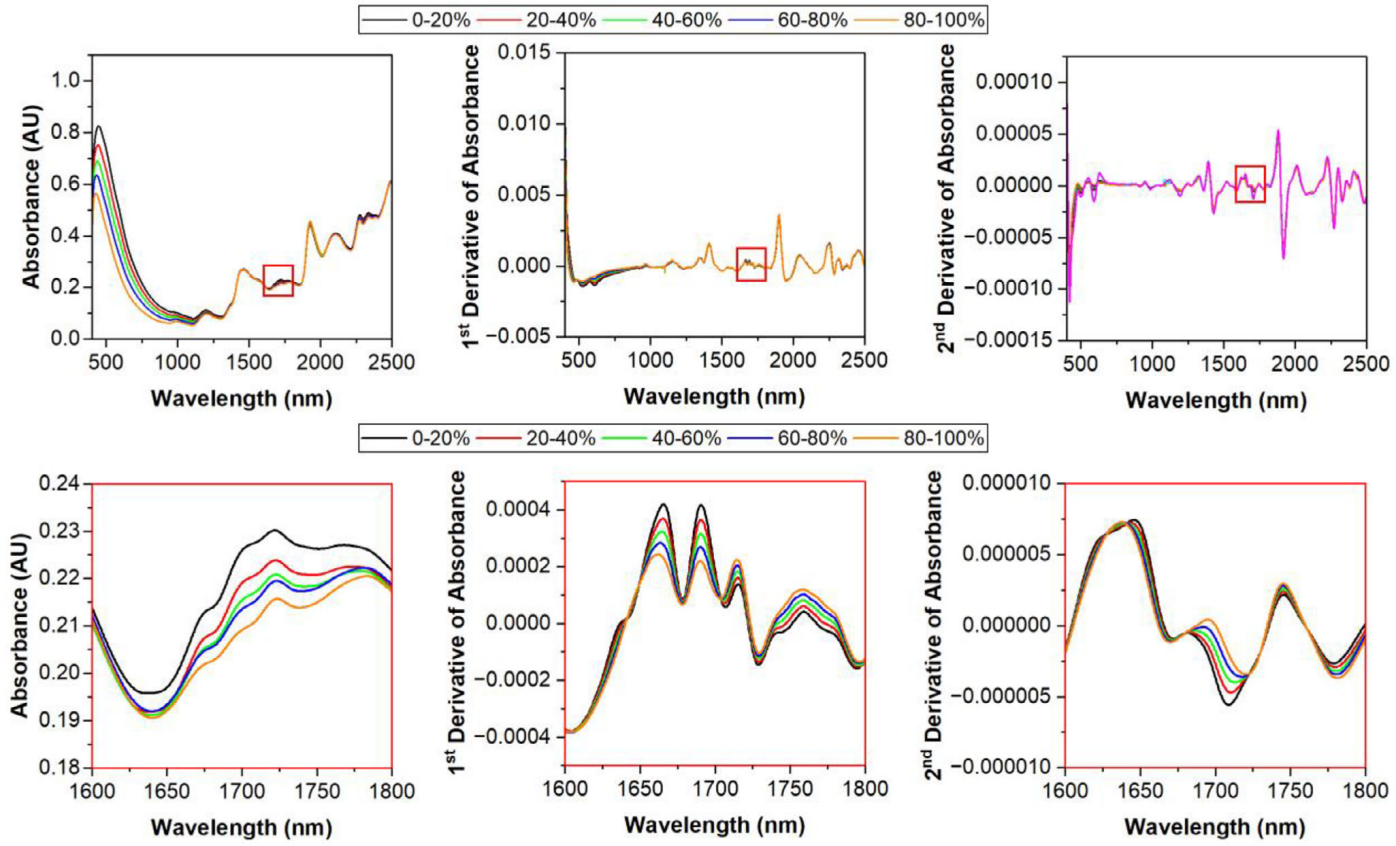
NIR spectra of sandalwood powder mixtures containing 0–100% SW. Increasing SW content progressively attenuates extractive‐related absorbance and enhances carbohydrate‐associated features, particularly after derivative preprocessing, enabling accurate HW‐SW ratio prediction.

The spectral manifestation of this compositional gradient is clearly captured in Figure 7, which shows raw and derivative spectra for powder mixtures spanning 0%–100% SW content. In raw absorbance spectra, the progressive attenuation of extractive‐associated features with increasing SW fraction is consistent with dilution of oil‐rich HW material. Simultaneously, carbohydrate‐associated spectral contributions become more pronounced.

Derivative preprocessing again enhances discriminative power, particularly in the 1600–1800 nm region, where extractive‐related absorbance diminishes systematically with increasing SW content. Unlike solid logs or chips, powder mixtures exhibit more linear, monotonic spectral trends, which explain the exceptionally high predictive performance observed in this application.

Figure 14 confirms near‐unity *R* ^2^ values for several HW‐SW powder models. The external‐validation parity plot in Figure 15 shows close alignment with the 1:1 line (*R* ^2^ = 0.99, RMSE = 0.77, MAE = 0.54), indicating low bias and variance.

Industrially, this application provides a powerful tool for quantifying SW contamination in milled material, enabling tighter control over feedstock quality and minimising unnecessary distillation of non‐productive biomass.

#### 3.4.4 Application IV: Moisture Prediction in Sandalwood Oil

Moisture content is a critical quality and stability parameter in Sandalwood oil, directly influencing oxidative degradation, hydrolytic reactions, storage behaviour, and downstream purification efficiency. Even trace moisture levels can significantly affect oil shelf life and distillation performance, necessitating precise, frequent monitoring. Traditional moisture‐determination techniques, such as Karl Fischer titration or gravimetric analysis, are accurate but destructive, reagent‐intensive, and poorly suited to high‐throughput or in‐line industrial monitoring.

**FIGURE 8:**
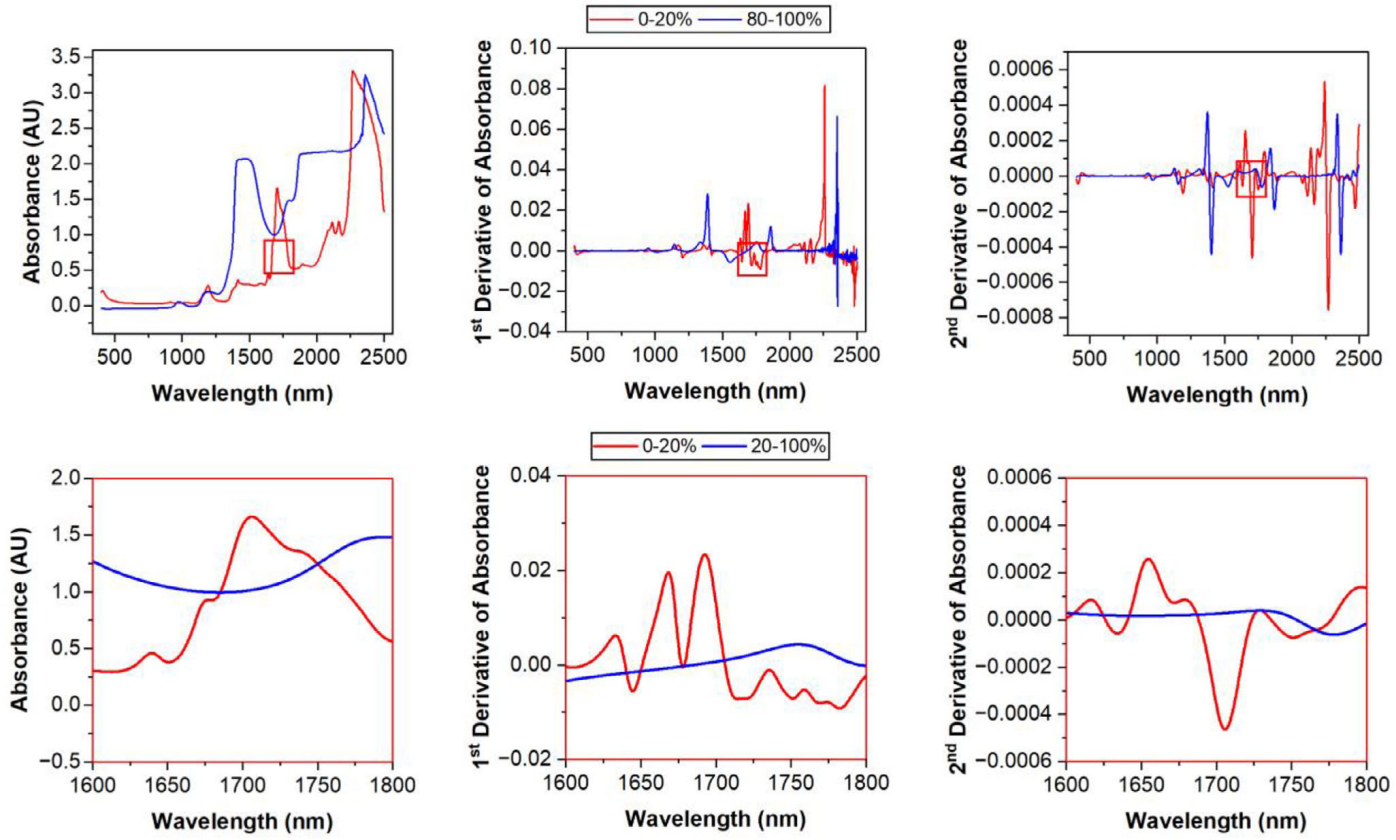
Raw and derivative NIR spectra of sandalwood oil across moisture levels. Moisture‐related O─H features remain evident after preprocessing and support quantitative prediction.

The spectral basis for moisture prediction is fundamentally distinct from that of yield or composition and is strongly governed by O─H vibrational overtones. This is clearly demonstrated in Figure 8, where the raw absorbance spectra of pure Sandalwood oil, grouped by moisture content, show pronounced intensity changes in the O─H‐dominated regions. Unlike solid matrices, liquid oil shows minimal scattering, allowing moisture‐related absorbance features to be clearly observed even in raw spectra.

Derivative preprocessing enhances spectral sensitivity to moisture by accentuating moisture‐specific features while reducing baseline effects (Figure 8). In the model benchmark, AdaBoost achieved a test‐set *R* ^2^ of 0.90 (Figure 14); external validation yielded *R* ^2^ = 0.86, RMSE = 0.19, and MAE = 0.10 (Figure 15).

However, model performance is intrinsically linked to the oil's physicochemical state. Variations in distillation methodology (batch, continuous, redistillation or CID) alter moisture distribution, phase behaviour, and trace compositional interactions, which can introduce additional spectral variance beyond that represented in the calibration set. Such process‐driven variability represents a key source of performance degradation and highlights the importance of calibration representativeness in industrial deployment.

Despite these challenges, the parity plot in Figure 15 (moisture) demonstrates close agreement between predicted and reference values, with RMSE and MAE remaining within practical industrial tolerance ranges. These results confirm that NIRS can provide reliable, non‐destructive moisture monitoring, provided that calibration datasets adequately capture process variability.

From an industrial perspective, this application enables real‐time, non‐destructive moisture monitoring during oil purification, storage, and blending, and can aid in detecting water‐related adulteration. It therefore reduces reliance on laboratory assays and supports early intervention to prevent quality degradation.

#### 3.4.5 Application V: Prediction of Ethanol Extract Yield

**FIGURE 9:**
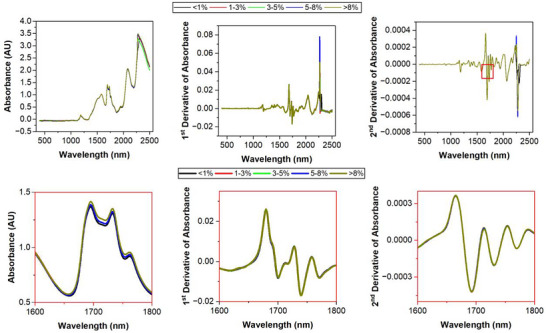
Raw and derivative NIR spectra of ethanol extracts grouped by extract yield. Derivative preprocessing suppresses the dominant solvent baseline and reveals reproducible solute‐related features.

Ethanol extraction offers a rapid, safer alternative to hexane extraction for assessing oil‐bearing potential; however, conventional workflows still require solvent evaporation and gravimetric quantification, which introduce delays and additional handling steps. Direct prediction of extract yield from liquid ethanol extracts using NIRS therefore offers an opportunity to streamline pre‐distillation screening and accelerate process decisions. This application is challenging because ethanol has strong O─H absorption that can mask solute‐related features. As shown in Figure 9, raw spectra across extract‐yield classes overlap substantially and show limited visual separation.

Derivative preprocessing is essential in this context. As shown in Figure 9, first and second derivatives suppress the ethanol baseline and amplify subtle spectral distortions associated with dissolved oil components. These derivative features, although small in magnitude, are consistent and chemically meaningful, enabling regression models to learn yield‐related patterns.

Figure 14 shows that linear and regularised models, particularly linear regression and ridge regression, performed strongly for ethanol extract yield. This result indicates that, after preprocessing, the solvent‐dominated spectra retained a systematic relationship with dissolved oil content. External validation in Figure 15 yielded *R* ^2^ = 0.98, RMSE = 0.53, and MAE = 0.24.

The strong external agreement supports solvent‐phase NIRS as a rapid proxy for extract yield. Industrially, this application enables processors to screen raw material without committing every sample to prolonged distillation, thereby improving laboratory throughput and resource allocation.

#### 3.4.6 Application VI: Portable NIRS Prediction of CID‐Emulsion Yield

Among all applications investigated, yield prediction from CID‐derived oil‐water emulsions represents the most analytically and operationally demanding use case. CID technology generates fine emulsions characterised by high turbidity, a variable droplet‐size distribution, and strong scattering effects, all of which complicate optical spectroscopy. Furthermore, this application relies on portable NIRS instrumentation that operates over a narrower spectral range (900–1700 nm) than benchtop systems.

**FIGURE 10:**
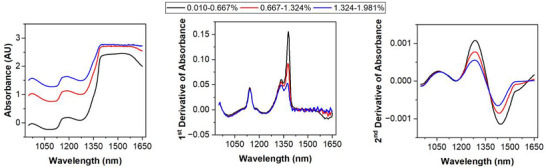
Raw and derivative NIR spectra of CID‐derived oil‐water emulsions. Derivative preprocessing reduces baseline distortion and retains features suitable for portable yield prediction.

The spectral characteristics of CID emulsions are shown in Figure 10. Raw absorbance spectra are dominated by scattering‐induced baseline distortion and water‐related absorption, limiting direct interpretation of the unprocessed data. Derivative preprocessing partially mitigates scattering effects by emphasising localised spectral curvature and relative changes. These features capture combined chemical and physical information related to oil‐droplet concentration, emulsion stability, and water‐oil interactions, effectively encoding a process fingerprint rather than a purely chemical signature.

Figure 14 demonstrates that nonlinear models, especially the deep multilayer perceptron, outperformed linear approaches for emulsion yield. The external‐validation parity plot in Figure 15 confirms strong agreement (*R* ^2^ = 0.94, RMSE = 0.28, MAE = 0.09), supporting real‐time process guidance.

This application demonstrates the feasibility of portable NIRS for complex, scattering‐dominated process streams. By enabling rapid yield estimation directly from CID emulsions, the approach can support closed‐loop optimisation of CID operating conditions and reduce reliance on the 5 h HD reference assay used for calibration.

#### 3.4.7 Application VII: Classification of HW, SW, Bark, and Transition Wood

Accurate classification of sandalwood material types is important for automated sorting, reduced transport of low‐value fractions, and improved process efficiency. Transition wood is especially challenging because it can visually resemble HW while containing little or no recoverable oil.

**FIGURE 11:**
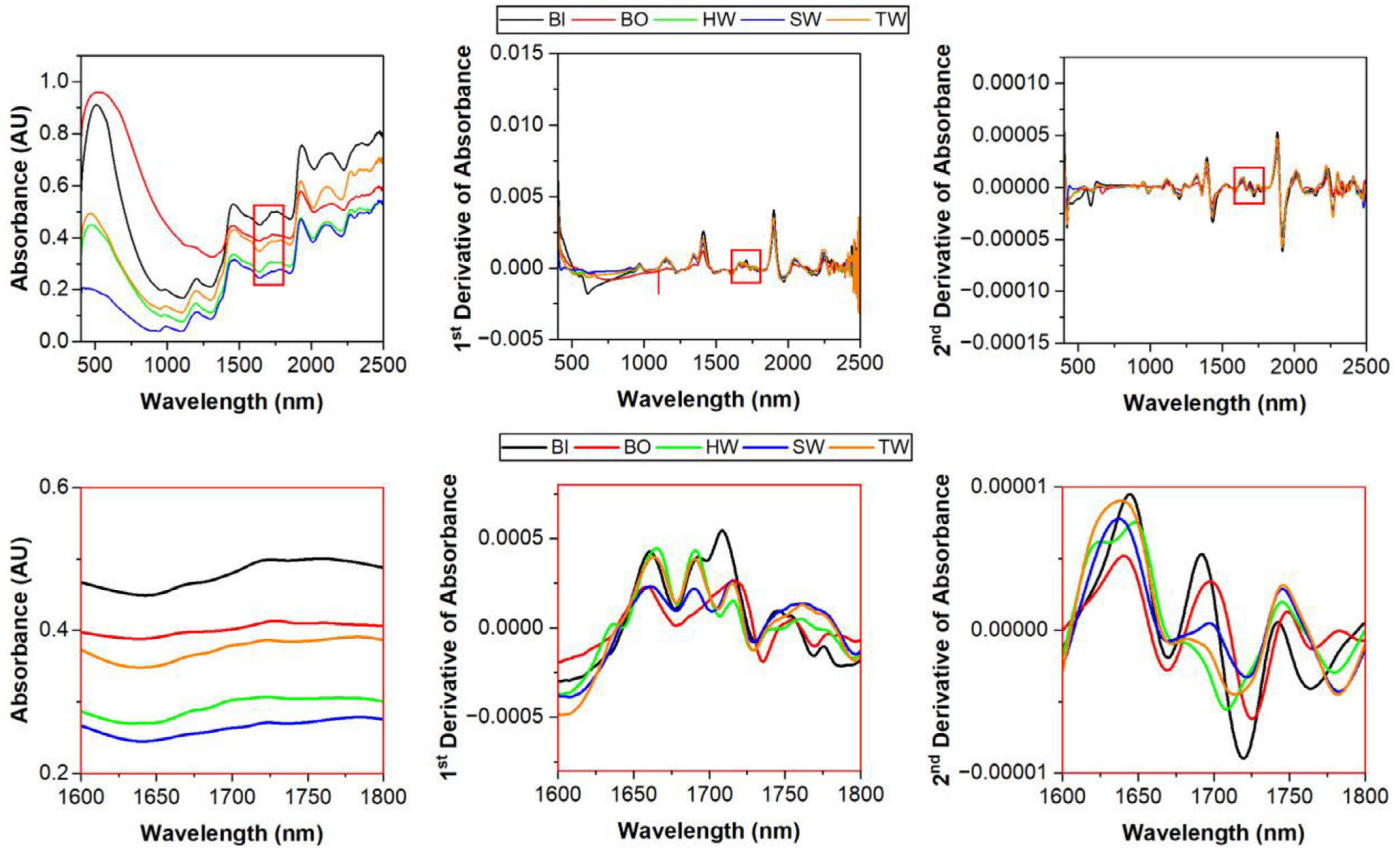
Raw and derivative NIR spectra of BI, BO, HW, SW, and TW. Derivative spectra in the 1600–1800 nm region reveal class‐specific patterns that are less apparent in raw absorbance spectra and support high‐accuracy classification.

The spectral basis for classification is explored in Figure 11, which presents raw and derivative spectra for BI, BO, HW, SW, and TW. Raw absorbance spectra exhibit partial overlap, reflecting the limitations of visual inspection and simple thresholding. However, derivative spectra, particularly in the 1600–1800 nm region, reveal class‐specific patterns associated with differences in extractive content, lignin composition, and carbohydrate abundance.

The global benchmark in Figure 14 shows high classification performance, with AdaBoost achieving the highest score. Figure 13 further indicates that derivative preprocessing improves class separability by suppressing baseline variability and enhancing chemically relevant features.

**FIGURE 12:**
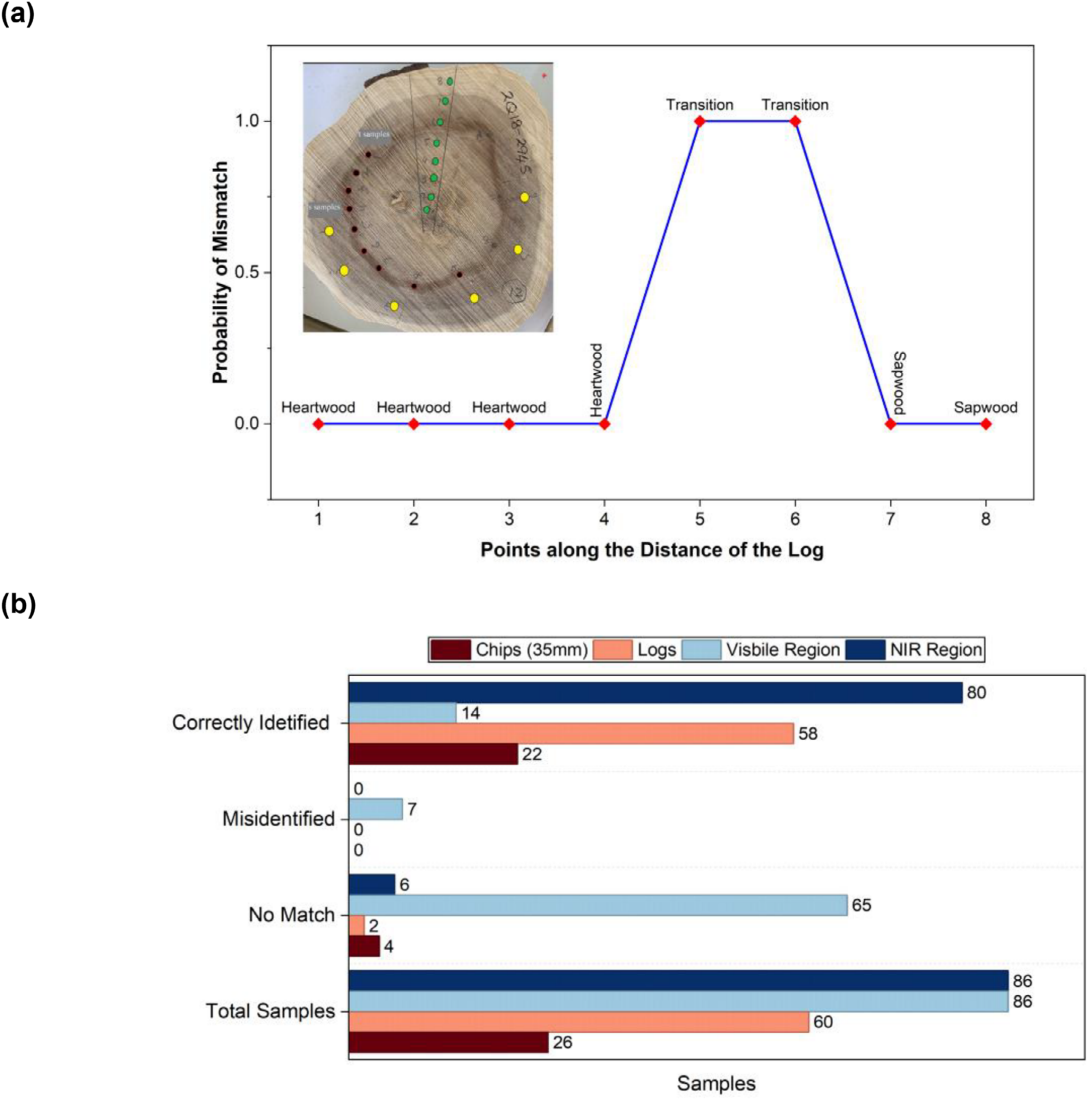
(a) Probability of spectral mismatch along the radial distance from pith to bark. Eight sampling positions are marked in the inset cross‐section; yellow and black points denote SW and TW samples, respectively. (b) Identification outcomes for 35 mm chips, whole logs, and visible‐ vs. NIR‐region spectra, reported as correct identifications, misidentifications, and no matches. NIR measurements provided the highest identification performance.

Ground truth validation and spatial complexity are addressed in Figure 12, which combines photographic documentation of classified samples with mismatch probability analysis across radial sampling points in log cross‐sections. Elevated mismatch probability in transition zones highlights the intrinsic ambiguity of this class and underscores the need for spectroscopic classification over manual sorting.

From an industrial standpoint, this application enables objective, scalable material segregation, reducing the transport and processing of non‐productive SW and bark and improving distillation efficiency.

##### 3.4.7.1 Ground‐Truth Classification and the Transition‐Wood Challenge

Figure 12 provides spatially resolved evidence for classification uncertainty and shows why transition wood is a critical industrial challenge.

Figure 12a shows the radial distribution of classification mismatch across a sandalwood log cross‐section. Mismatch probability remains low within homogeneous HW and SW domains but increases sharply within transition zones. This spatial pattern indicates that classification error is correlated with anatomical gradients and extractive‐content heterogeneity rather than being purely stochastic. Transition wood represents an intermediate physicochemical state in which extractive concentration, lignin composition, and carbohydrate fractions vary continuously. Its spectral signatures therefore overlap with those of adjacent wood types, producing intrinsic class ambiguity.

Figure 12b presents classification results across different acquisition domains and sample types, showing that NIR‐based classification reliably surpasses visible‐region classification. The NIR domain, which captures overtone and combination absorptions of C─H and O─H vibrations associated with extractives and structural polymers, yields higher correct‐identification rates and markedly reduced misidentification and “no match” frequencies relative to visible‐only features. This confirms that chemically sensitive spectral information provides greater discriminative power than morphology‐dependent visual cues, particularly in boundary regions where anatomical transitions are gradual. This figure strengthens the industrial argument by demonstrating that the classification challenge is not theoretical; it is spatially localised, systematic, and economically consequential.

Together, these applications demonstrate that NIRS, coupled with AI/ML, can support rapid yield prediction, compositional quality control, moisture monitoring, extraction forecasting, CID‐emulsion assessment, and automated material sorting, thereby reducing time, energy use, waste, and economic uncertainty in industrial sandalwood processing.

### 3.5 Effects of Preprocessing on Model Performance

**FIGURE 13:**
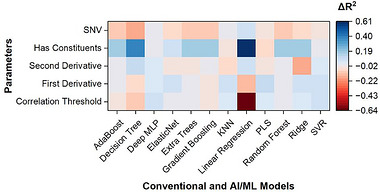
Δ*R* ^2^ associated with SNV, first‐ and second‐derivative preprocessing, constituent inclusion, and correlation thresholding across conventional and AI/ML models. Ensemble methods were comparatively robust, whereas linear models were more sensitive to preprocessing changes.

Model performance resulted from systematic optimisation of preprocessing‐model interactions. Figure 13 summarises Δ*R* ^2^ values across SNV, derivative, correlation‐threshold, and model configurations.

The heatmap shows that ensemble models are comparatively robust to preprocessing changes because they can select informative features and accommodate nonlinearities. Linear and latent‐variable models are more sensitive to shifts in the variance structure and to baseline or scattering artefacts. SNV and derivative transformations frequently improved performance for high‐scatter matrices, consistent with Table 1 and the application‐specific results.

### 3.6 Cross‐Application Model Benchmarking

**FIGURE 14:**
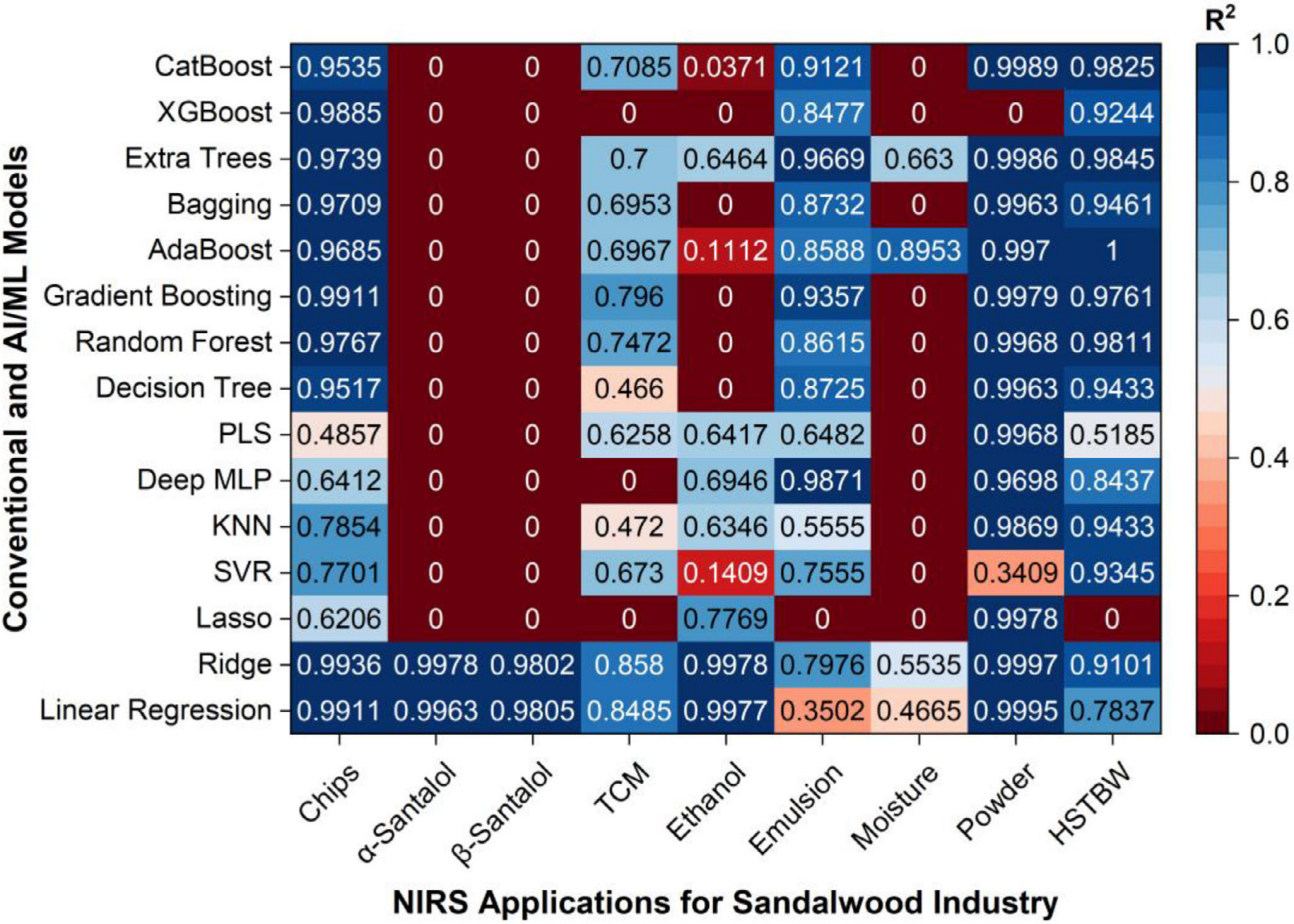
*R* ^2^ heatmap for conventional chemometric and AI/ML models across NIRS applications. Linear and regularised models performed best for several yield and composition tasks, whereas nonlinear and ensemble approaches were strongest for emulsion, moisture, and classification tasks.

Figure 14 summarises *R* ^2^ values across models and applications. Performance was application‐dependent: linear and regularised models were strongest for chip yield, santalols, ethanol extract yield, and powder ratio, whereas nonlinear or ensemble methods were favoured for emulsion yield, moisture, and wood‐type classification. The comparison therefore supports a multi‐model portfolio rather than the universal dominance of a single algorithm family.

Figure 14 thus supports the central conclusion that the optimal model depends on matrix chemistry, optical complexity, and deployment context.

### 3.7 External Validation of the Best‐Performing Models

**FIGURE 15:**
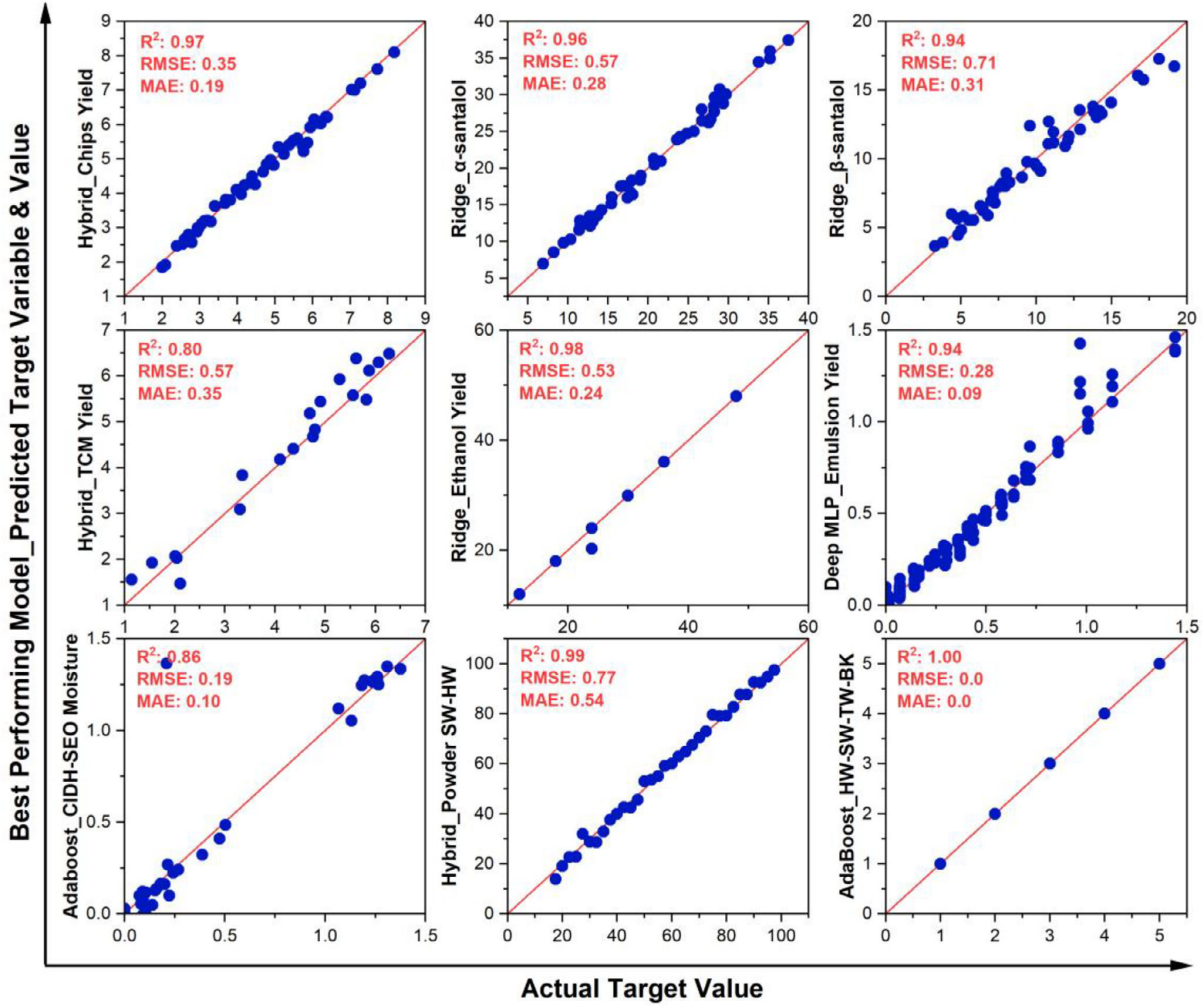
External‐validation parity plots for the best‐performing models: chip yield, α‐santalol, β‐santalol, TCM yield, ethanol extract yield, CID‐emulsion yield, moisture, powder HW‐SW ratio, and wood‐type classification. *R* ^2^ values ranged from 0.80 to 1.00.

Figure 15 presents predicted‐vs.‐measured parity plots for the best‐performing model in each application, with *R* ^2^, RMSE, and MAE for physically independent external‐validation samples. Most targets show close agreement with the 1:1 line. TCM yield (*R* ^2^ = 0.80) and oil moisture (*R* ^2^ = 0.86) were comparatively more difficult than chip yield, santalol, ethanol extract yield, emulsion yield, powder ratio, and classification.

These results support the “best model per application” strategy. As described in Section 2.7, validation was independent at the physical‐sample level; because harvest year and plantation were not universal hold‐out factors, the plots should not be interpreted as formal cross‐year or cross‐plantation transfer tests.

### 3.8 Error Structure and Robustness Beyond *R* ^2^

**FIGURE 16:**
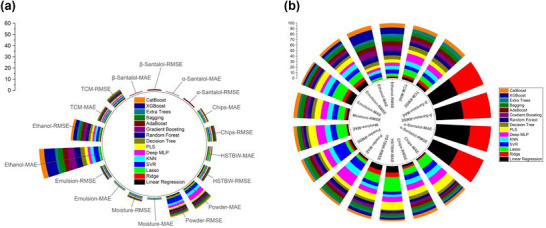
Radial plots of (a) MAE and (b) RMSE for each model‐application combination. Error magnitudes should be compared primarily within each target because the applications use different measurement scales.

Figure 16 compares MAE and RMSE across model‐application combinations.

Figure 16a shows that the error magnitude depended on both the target scale and the matrix complexity. Moisture and powder‐ratio tasks produced relatively compact error profiles, whereas some solvent and heterogeneous solid‐matrix tasks showed larger dispersion. Because MAE values are expressed in target‐specific units, within‐application comparisons among models are more informative than direct comparisons across targets.

Within applications, nonlinear and ensemble methods often reduced error for complex matrices, whereas linear and regularised methods remained competitive or superior for chemically structured tasks such as santalol and ethanol extract prediction. This pattern is consistent with Figure 14 and reinforces application‐specific model selection.

Figure 16b presents RMSE, which weights larger deviations more strongly and highlights sensitivity to outliers. The relative ranking of models again varied by application; no single family minimised error across all targets. Agreement between MAE and RMSE trends within a task provides an additional indication of model stability.

Overall, robustness arose from matching model complexity to matrix behaviour rather than from the systematic superiority of ensemble methods.

### 3.9 Cross‐Application Insights and Analytical Implications

Taken together, Applications I‐VII show that predictive performance depends on chemical signal strength, matrix‐driven optical complexity, preprocessing, and model behaviour. Chemically structured tasks such as chip yield, santalol composition, ethanol extract yield, and powder HW‐SW ratio were effectively modelled using linear or regularised approaches after appropriate preprocessing. Moisture, CID emulsions, and classification benefited more from nonlinear or ensemble methods. These results demonstrate that the choice of algorithm must be matched to the matrix and target rather than prescribed in advance.

CID emulsions illustrate the challenge posed by strong scattering and multiphase structure, whereas transition wood illustrates overlapping class boundaries. In these settings, nonlinear models provide additional flexibility for interactions among absorbance, scattering, and compositional covariance. By contrast, the strong performance of ridge and linear regression for several yield and composition tasks shows that model complexity alone does not determine accuracy.

Qualitative classification followed the same principle. Well‐separated classes can be captured by simpler decision boundaries, whereas transition wood requires models that accommodate gradual anatomical and chemical gradients. Ensemble classifiers therefore offer particular value at class boundaries, where correlated spectral features and nonlinear overlap reduce the reliability of visual or threshold‐based sorting.

The Δ*R* ^2^ analysis supports this interpretation: ensemble models were comparatively invariant to preprocessing choices, whereas linear models were more sensitive to changes in baseline and variance structure [26, 53]. Sensitivity does not imply inferiority; however, in spectrally well‐conditioned applications, linear and regularised models produced the highest predictive accuracy. Preprocessing and algorithm selection should therefore be optimised jointly.

These matrix‐dependent trends are consistent with previous NIRS studies. Moisture‐dominated systems often exhibit strong, relatively direct spectral responses [54], while derivative preprocessing can improve performance in heterogeneous wood matrices [55]. Kernel and ensemble methods are useful when relationships become nonlinear [56], although model complexity should remain matched to the application rather than assumed to be universally beneficial [57].

The Δ*R* ^2^ analysis provides quantitative evidence for this principle [12]. Ensemble models were comparatively robust to preprocessing variation, whereas linear and latent‐variable methods were more sensitive when scatter‐related transformations altered the variance structure [58]. More broadly, algorithmic robustness is a central consideration when translating AI‐assisted spectroscopy from controlled modelling studies to operational measurement systems [59].

A second cross‐cutting insight is the partial decoupling of oil yield and composition. Because high yield does not guarantee a high santalol fraction, harvest valuation, blending, distillation control, and quality assurance require separate predictive targets rather than a single proxy. Accordingly, spectroscopy functions not merely as a measurement tool but as a decision‐support architecture in which application‐specific models provide targeted information across processing stages [60].

The results also clarify the physical limits of optical prediction. Performance reductions in scattering‐dominated emulsions and elevated uncertainty in transition wood zones arise from intrinsic material heterogeneity rather than modelling deficiency. Spectroscopic AI systems, therefore, operate probabilistically, delivering bounded‐confidence predictions that must be interpreted within process‐aware decision frameworks [32, 61].

From a broader perspective, the study contributes to the integration of spectroscopy with AI/ML. Classical chemometric methods remain effective when spectral‐target relationships are approximately linear [62], whereas ensemble approaches can provide robustness in complex optical environments [63]. By benchmarking both across matrices, this work establishes a context‐aware modelling strategy for heterogeneous biological resources.

Industrial implications extend beyond sandalwood [11, 64]. The framework illustrates how spectroscopy, combined with structured preprocessing evaluation, model benchmarking, and external validation, can serve as a scalable analytical backbone for bioresource industries. Such systems enable rapid, non‐destructive assessment of yield, quality, and process state, reduce reliance on time‐intensive laboratory assays, and support more efficient, data‐driven operations.

These cross‐application results also clarify the scope of external validation. The physically independent hold‐out sets reduce the risk of spectral leakage, but harvest year, plantation, tree, and processing batch were not imposed as universal domain hold‐out factors across all applications. Future calibration‐transfer studies should therefore test performance prospectively across plantations, harvest years, instruments, and operating environments.

## 4 Conclusion

This study establishes a unified NIRS‐AI framework to replace or reduce destructive laboratory assays throughout the *S. album* processing value chain. By integrating benchtop (400–2500 nm) and portable (900–1700 nm) spectroscopy with systematic preprocessing, feature engineering, and model benchmarking, the platform demonstrated predictive capability across solid wood, powders, oils, solvent extracts, and emulsions.

On physically independent external‐validation samples, EO yield from solid matrices achieved *R* ^2^ = 0.97; α‐ and β‐santalol achieved *R* ^2^ = 0.96 and 0.94; powder HW‐SW ratio achieved *R* ^2^ = 0.99; moisture in oil achieved *R* ^2^ = 0.86; ethanol extract yield achieved *R* ^2^ = 0.98; and portable CID‐emulsion yield achieved *R* ^2^ = 0.94. Wood‐type classification also showed high accuracy, with NIR discrimination outperforming visible‐region identification in transition zones. These results support rapid pre‐distillation valuation, compositional quality assessment, moisture monitoring, extraction screening, and material sorting.

Across applications, no single algorithm family was universally optimal. Linear and regularised models performed best for several chemically structured yield and composition tasks, whereas nonlinear or ensemble models were strongest for moisture, emulsion, and classification tasks. Δ*R* ^2^ analysis further showed that ensemble models were comparatively robust to preprocessing variation, while linear models required more careful control of baseline and variance structure.

Collectively, these findings establish NIRS‐AI as a scalable analytical backbone for high‐value bioprocessing. By enabling rapid, non‐destructive assessment of yield, quality, moisture, extraction potential, and material class, the framework supports real‐time decision‐making, reduces reliance on prolonged distillation and chromatographic assays, and improves resource efficiency. Because harvest year and plantation were not universal hold‐out factors, further prospective transfer studies are warranted before broad deployment across new plantations and seasons.

## Author Contributions

**Muhammad Hassnain**: writing – original draft, writing – review & editing, software, methodology, formal analysis, data curation, conceptualisation. **Muhammad Rizwan Azhar**: writing – review & editing, supervision, resources, project administration.

## Conflicts of Interest

The authors declare no conflict of interest.

## Authors’ Declaration

This research paper has been prepared by the authors in their personal capacities based on scientific and technological knowledge and expertise. The views, opinions, interpretations, and conclusions expressed in this work are solely those of the authors and do not reflect the views, positions, or policies of any current or former employers, organisations, governmental bodies, or affiliated institutions. The content was developed independently and was not influenced by any professional roles or responsibilities held by the authors. Any resemblance to actual events, policies, data, assets, places or entities is purely coincidental, and no part of this work should be construed as representing or implicating any institution with which any of the authors are, or have been, affiliated.

## Declaration of Generative AI and AI‐Assisted Technologies in the Writing Process

During preparation and revision of this work, the authors used Grammarly to improve language, organisation, and readability. These tools were not used to generate or analyse data, perform statistical modelling, or draw scientific conclusions. The authors reviewed and edited all AI‐assisted text and take full responsibility for the content of the manuscript.

## Data Availability

The data supporting the findings of this study are owned by Quintis Sandalwood Pty Ltd. Due to commercial confidentiality, the data are not publicly available but may be made available upon reasonable request, subject to permission from Quintis Sandalwood Pty Ltd.
